# Stigma and health inequality experienced by ethnic minorities during the COVID-19 pandemic in a Chinese community: an implication to health policymakers

**DOI:** 10.3389/fpubh.2023.1184209

**Published:** 2023-05-25

**Authors:** Judy Yuen-Man Siu, Yuan Cao, David H. K. Shum

**Affiliations:** ^1^Department of Applied Social Sciences, Faculty of Health and Social Sciences, The Hong Kong Polytechnic University, Hong Kong, Hong Kong SAR, China; ^2^Interdisciplinary Centre for Qualitative Research and Training, The Hong Kong Polytechnic University, Hong Kong, Hong Kong SAR, China; ^3^International Research Centre for the Advancement of Health Communication, The Hong Kong Polytechnic University, Hong Kong, Hong Kong SAR, China; ^4^Research Centre for SHARP Vision, The Hong Kong Polytechnic University, Hong Kong, Hong Kong SAR, China; ^5^Department of Rehabilitation Sciences, Faculty of Health and Social Sciences, The Hong Kong Polytechnic University, Hong Kong, Hong Kong SAR, China; ^6^Research Institute for Intelligent Wearable Systems, The Hong Kong Polytechnic University, Hong Kong, Hong Kong SAR, China; ^7^Research Institute for Smart Ageing, The Hong Kong Polytechnic University, Hong Kong, Hong Kong SAR, China; ^8^Mental Health Research Centre, The Hong Kong Polytechnic University, Hong Kong, Hong Kong SAR, China

**Keywords:** ethnic minorities, stigma, social segregation, structural disparities, COVID-19, Hong Kong

## Abstract

**Introduction:**

Ethnic minorities are considered one of the most vulnerable groups during the COVID-19 pandemic. However, the explanatory pathway of how their disadvantaged experiences during epidemics are related to the embedded and longstanding stigmas against them and how these embedded stigmas can affect their resilience in disease outbreaks are not well understood. This study investigated the experiences of ethnic minorities in the COVID-19 pandemic, and how their experiences were related to the embedded stigma toward them.

**Methods:**

This study adopted a qualitative approach, interviewed 25 individuals (13 women and 12 men) from ethnic minority groups residing in Hong Kong from August 2021 to February 2022 in a semi-structured format. Thematic analysis was conducted to analyze the data.

**Results:**

The participants were isolated and stereotyped as infectious during the COVID-19 pandemic at community and institutional levels. Their experiences did not occur suddenly during the pandemic but were embedded in the longstanding segregation and negative stereotypes toward ethnic minorities in different aspects of life before the pandemic. These negative stereotypes affected their resilience in living and coping with the pandemic.

**Conclusion:**

The participants’ experiences during the COVID-19 pandemic were mostly disadvantageous and predominantly initiated by the mainstream stigmatization toward them by the local Chinese residents and government. Their disadvantaged experiences in the pandemic should be traced to the embedded social systems, imposing structural disparities for ethnic minorities when accessing social and medical resources during a pandemic. Because of the preexisting stigmatization and social seclusion of ethnic minorities in Hong Kong, the participants experienced health inequality, which stemmed from social inequality and the power differential between them and the Chinese locals. The disadvantaged situation of the participants negatively affected their resilience to the pandemic. To enable ethnic minorities better cope with future epidemics, merely providing assistance to them during an epidemic is barely adequate, but a more supportive and inclusive social system should be established for them in the long run.

## 1. Introduction

Ethnic minorities comprised 8% of the total population in Hong Kong as of 2017 (1). After excluding foreign domestic helpers, ethnic minorities accounted for 3.6% of the entire Hong Kong population, of which people from South Asia–mostly the Indians and Pakistanis–were the largest ethnic minority group and comprised 14.5% of the ethnic minority population in Hong Kong (1). Ethnic minorities had a higher labor force participation rate (85.9%) than that of the entire population (60.8%) (1). The majority of the working ethnic minorities (74.7%) were engaged in elementary and nonprofessional occupations, and their median monthly income was HK$20,000 (equivalent to US$2,564) (1). Besides the South Asians, there were 3,144 Africans living in Hong Kong in 2016 (2).

South Asians encounter more difficulties than do other ethnic minority groups in Hong Kong and are often described as the poorest residents in the city (3). Their labor force participation rate was 79.4 and 53.4% for men and women, respectively, but they have the lowest median monthly income among all ethnic minorities in Hong Kong excluding foreign domestic helpers (1). Also, a gender pay gap exists among them, and the median monthly incomes for men and women were HK$16,200 (equivalent to US$2,077) and HK$9,750 (equivalent to US$1,250), respectively (1). South Asian minorities are mostly employed in elementary occupations (30.2%) and the service and sales industry (26%) (1). The education level of South Asian minorities was also lower than that of the general population; only 39.7% attained upper secondary school education, and 33.5% attained postsecondary education (1).

Ethnic minorities in Hong Kong are considerably disadvantaged. Studies have revealed that the disadvantaged positions of ethnic minorities can exacerbate their difficulties during epidemics and ultimately worsen their health and social well-being (4–6). Therefore, ethnic minorities experience health inequality, which is correlated with social inequality (7), and face stigmatization and discrimination during disease outbreaks (8). However, migrant health is noted to be slow in moving upstream in policy development and literature, as ethnicity is not commonly accepted as an important social determinant in leading to health inequality (9). This study, therefore, fills the literature gap by investigating how ethnicity can serve as an important social determinant for contributing health inequality in a pandemic. Also, although studies have indicated that ethnic minorities often have poorer health outcomes in the COVID-19 pandemic (4–6, 10–12), the reasons for their often poorer health outcomes in the pandemic are not well documented. This study attempts to identify and discuss these reasons.

Two theories have been suggested to understand the disadvantaged situation of ethnic minorities. Competition theory traces the origins of ethnic conflict to the struggle between ethnic groups for scarce resources, resorting stigmatization and discrimination to economic reasons (13); but it fails to apply in developed societies where economic affluence and social security systems are notable. Also, how ethnic minorities respond to disadvantaged situations remains unknown in competition theory (14). Conflict theory, on the other hand, explains the ethnic and racial dynamics of the social power exercised by the majority group over ethnic minorities (15). Ethnic minorities can be labeled as “dangerous” by mainstream groups, which results in racial profiling and further stigmatization (16). According to the conflict theory, the stigmatization and vulnerability of ethnic minorities during the COVID-19 pandemic should be understood as a social process embedded in the power differentials between ethnic minorities and majority groups in a society. However, few studies have investigated the explanatory pathway of this embedded social process or the relationship between the experiences of ethnic minorities during the COVID-19 pandemic and the social experiences before the pandemic. Following the conflict theory as the framework, this study was conducted to demonstrate the explanatory pathway of how the experiences of ethnic minorities in Hong Kong during the COVID-19 pandemic are embedded in the longstanding power differentials between ethnic minorities and majority groups, and how this can affect their resilience in disease outbreaks.

## 2. Methods

### 2.1. Ethical considerations

Ethics approval was obtained from the Human Subjects Ethics Subcommittee of the Hong Kong Polytechnic University before the study began (ID: HSEARS20200827001-04). All the procedures were performed in accordance with the relevant guidelines and regulations. The participants were informed of the study procedures before they were interviewed, and informed consent was obtained and recorded from all participants. To protect participant privacy, no personal information was collected in the audio or coded data. All the data were stored in password-protected files, and interview transcripts were coded with anonymous identifiers to protect participant privacy. After the interviews were transcribed, the audio recordings were destroyed.

### 2.2. Data collection

A qualitative approach adopting individual, semi-structured, in-depth interviews was conducted with 25 individuals (13 women and 12 men) aged 18 years or older from ethnic minority groups in Hong Kong from August 2021 to February 2022. They were recruited using purposive sampling based on the following criteria: they (1) were aged 18 years or older, (2) were not of Chinese ethnicity, and (3) lived in Hong Kong prior to January 2020, when the COVID-19 pandemic began to spread in Hong Kong. These criteria were formulated to ensure that the participants were exposed to Hong Kong society not only during the COVID-19 pandemic but also before the pandemic to investigate how the ethnic experiences during the pandemic were embedded in and transmitted from mainstream society before the pandemic.

Of the 25 participants, seven were recruited from a local university and were postgraduate students (*n* = 6) or staff (*n* = 1). The remaining 18 participants were recruited from three other sources to diversify the socioeconomic background of the research participants: the Hong Kong Christian Service (a non-governmental organization), a district councilor office in Hong Kong, and snowball sampling. The Hong Kong Christian Service has a team that offers social services to disadvantaged ethnic minorities (17). The assistant of a district councilor’s office also worked for the Hong Kong Christian Service and worked with ethnic minority groups. The Hong Kong Christian Service and district councilor assistant assisted with sampling in accordance with the sampling criteria. Snowball sampling involved recruiting participants referred by previous participants. The research assistant conducted further screening for these potential participants and invited them to participate in interviews if they fulfilled the sampling criteria.

The recruitment of the participants was stopped when data saturation had been achieved, indicating no new themes or codes were emerged from further interviews (18). Data was saturated in the 23rd interview, and two more interviews were conducted to confirm data saturation.

To ensure that the interviews were administered consistently, the participants were interviewed individually by the same research assistant. The research assistant has a training background in anthropology at the Bachelor’s and Master’s levels, and toward the end of our study he was about to enroll as a PhD student in anthropology. He has experience in researching ethnic minorities in Hong Kong. To ensure the quality of the interviews, the research assistant received intensive interview training from the first author. The first author has a training background in anthropology at the Bachelor’s and Master’s levels and in medical anthropology at the research doctorate level (Ph.D). Both the trainer and trainee of the interviews in this study have extensive experience in qualitative research and interviews. All the authors supervised the research assistant throughout the data collection process and provided guidance. Twenty-four interviews were conducted in English, which was the language that could be spoken by both the interviewer and participants. One interview was conducted in Cantonese, which was the native tongue of the interviewer and one participant who was born and educated in Hong Kong. Selected interview quotes from this interview used in reports were translated from Cantonese to English by the interviewer.

An interview question guide with an inductive design was used to guide the interview discussions. The interview question guide was developed with reference to the literature on the experiences of ethnic minority groups during the COVID-19 pandemic and other pandemics (4, 6, 8) and on fieldwork observations of ethnic minorities. The questions addressed the participants’ experiences before and during the COVID-19 pandemic to investigate the interrelationship between the triggers and underlying reasons of their experiences. To offer the participants flexibility in telling their stories and experiences, the questions were open-ended (19).

Because the interviews were conducted during the ongoing COVID-19 pandemic, all 25 interviews were conducted online over Zoom or WhatsApp video call. The interviewer interviewed the participants in a private room at the authors’ institution to protect participants’ confidentiality. Each interview lasted between 40 min and 1.25 h and was audio-recorded with the participant’s consent. As compensation for their time, each participant was given a supermarket cash coupon worth HK$200 (approximately US$26) upon completion of the interview.

### 2.3. Data analysis

The interviewer wrote fieldnotes immediately after each interview to identify critical data points. The interviews were transcribed verbatim, and a thematic analysis approach was used to investigate the meaning and essence of the participants’ lived experiences (20). The first author and research assistant (interviewer) conducted data analysis independently by bracketing their presuppositions. All transcripts were read for content familiarization and getting a sense of the whole experience of the participants, and reread to determine possible themes (20). The interview transcripts were then analyzed line-by-line through an inductive coding process to enable the identification of the participants’ thinking and behavioral patterns (20). All significant statements or phrases that are of relevance to the participants’ unpleasant and stigma experiences and the meanings relevant to the phenomenon were identified, extracted, descripted, and broken down into codes, which were labeled and then collated into categories (20). Recurrent categories were highlighted. Overlapping codes and categories were consolidated to form broader themes after repeated examination and comparison (20). The codes, categories, and themes derived from the data, alongside supporting interview quotes, were documented in a coding table (20), where designated concepts and categories were highlighted to translate the interviews into meaningful symbols to enable understanding of the participants’ thoughts.

Data analysis meetings were held every 2 weeks so that the first author and research assistant (interviewer) could discuss the data and reach a consensus in interpreting the participants’ experiences. Also, meetings were organized monthly so that all the authors could discuss about the findings from time to time. The finalized results were agreed upon by the second and third authors.

### 2.4. Data trustworthiness

Data collection and analysis were conducted according to the guidelines for the Consolidated Criteria for Reporting Qualitative Research (21). The criteria developed by Lincoln and Guba were used to ensure the rigor of the study design and methods (22). Data saturation was achieved (18). The coding table includes the quotes from the interviews to ensure that the codes, categories, and themes were grounded in the interview data. Cross-checking between the interview quotations, codes, categories, and themes was performed throughout the analysis. The coding procedure was conducted independently by the first author and the research assistant (interviewer) who are experienced in qualitative analysis, and a consensus on the coding was reached.

## 3. Results

### 3.1. Participant characteristics

The participant demographics are summarized in Table 1. All the participants were ethnic minorities originally from South Asia or Africa who were residents of Hong Kong. Three, seventeen, and five participants were from India, Pakistan, and Ghana, respectively. The age range of the participants was 18–49 years, 21 participants were university-educated, and four participants completed secondary school. Three participants received social security assistance from the government, and two participants refused to disclose whether they received assistance. During the pandemic, eight participants were working full-time, three participants were working part-time, four participants were full-time homemakers, and one participant was unemployed. All the participants could read, write, and speak English. Four Pakistani participants were born in Hong Kong and could communicate verbally in Cantonese Chinese, one of which could read and write some Chinese. Most of the participants received at least one dose of the COVID-19 vaccination, one refused, and three were hesitant at the time of the study. One participant had a history of COVID-19 infection.

**Table 1:** Demographics of the participants.

Informant code	Gender	Age range	Ethnicity	Education level	Occupation	Monthly personal income (in HKD)	Types of social support assistance received	Marital status	Housing
176	F	35–39	South Asian, Hong Kong-Born Pakistani	Senior secondary	Part-time education	4,000–9,999	No	Married	Self-owned public housing
177	F	40–44	South Asian, Pakistani	Bachelor	Housewife	0	No	Married	Self-owned public housing
178	F	35–39	South Asian, Pakistani (in Hong Kong for 18 years)	Bachelor	Housewife	0	No	Married	Self-owned public housing
179	M	35–39	South Asian, Indian (in Hong Kong since 2001)	Senior secondary	Full-time trading	20,000–29,999	Working Family Allowance	Married	Rental private housing
180	F	20–24	South Asian, Hong Kong-born Pakistani	Bachelor	Full-time education	20,000–29,999	No	Single	Rental public housing
181	M	40–44	South Asian, Pakistani	Senior secondary	Unemployed	0	Did not disclose	Married	Rental public housing
182	M	35–39	South Asian, Pakistani (in Hong Kong since 2018)	Master or above	Full-time research	20,000–29,999	No	Married	Rental private housing
183	F	20–24	South Asian, Hong Kong-born Pakistani	Bachelor	Part-time education	>50,000	No	Single	Rental public housing
184	F	35–39	South Asian, Pakistani	Bachelor	Full-time education	15,000–19,999	No	Married	Rental private housing
185	M	45–49	South Asian, Pakistani (in Hong Kong since 2011)	Master or above	Part-time interpreter	20,000–29,999	No	Married	Rental public housing
186	F	35–39	South Asian, Pakistani (COVID infection history)	Bachelor	Housewife	0	Comprehensive Social Security Assistance	Married	Rental public housing
187	F	18–19	South Asian, Pakistani	Bachelor	Student	0	No	Single	Rental public housing
188	F	25–29	South Asian, Pakistani	Master or above	Research student	15,000–19,999	No	Single	Others (unspecified)
189	F	45–49	South Asian, Indian (in Hong Kong since 1999)	Bachelor	Housewife	0	No	Married	Rental public housing
190	M	20–24	South Asian, Pakistani (in Hong Kong since 2011)	Bachelor	Full-time finance and insurance	30,000–39,999	No	Single	Rental public housing
194	F	30–34	South Asian, Indian (in Hong Kong since 2018)	Master or above	Full-time business related	0	No	Married	Rental private housing
240	M	25–29	South Asian, Hong Kong-born Pakistani	Bachelor	Full-time accounting	did not disclose	No	Single	Rental public housing
241	M	30–34	South Asian, Pakistani	Master or above	Research student	20,000–29,999	No	Single	Hostel
242	M	20–24	South Asian, Pakistani (in Hong Kong since 2018)	Bachelor	Full-time research assistant	15,000–19,999	No	Single	Rental private housing
247	M	25–29	African from Ghana (in Hong Kong since 2018)	Master or above	Research student	15,000–19,999	Did not disclose	Single	Hostel
248	M	25–29	African from Ghana	Master or above	Research student	15,000–19,999	No	Single	Hostel
249	F	30–34	African from Ghana	Master or above	Research student	15,000–19,999	No	Married	Hostel
250	M	40–44	African from Ghana	Master or above	Research student	20,000–29,999	No	Married	Hostel
253	M	25–29	African from Ghana	Master or above	Research student	15,000–19,999	No	Single	Hostel
254	F	18–19	South Asian, Hong Kong-born Pakistani	Senior secondary	Student	0	Comprehensive Social Security Assistance	Single	Rental public housing

Most of the participants lived in government-subsidized housing of public rental housing (*n* = 10) and self-owned public housing (*n* = 3). Six participants were living in subsidized student hostels, and five participants were living in private rental housing. None of the participants were living in self-owned private housing.

### 3.2. Experiences of the participants during the COVID-19 pandemic

The experiences of the participants during the COVID-19 pandemic in Hong Kong were closely tied to the power differentials, i.e., power differences, between themselves as minorities and the majority groups. Power differentials implies hierarchical order; those who are in the higher hierarchical order will have more power over those who are in the lower hierarchical order in a society or in an organization (23). Those who are in the lower hierarchical order tend to experience the negative effects from such power relationship, and they are in a more vulnerable position of being stereotyped (23). For such power differential, the participants–as ethnic minorities–were those who are in the lower hierarchical order that always felt marginalized at the community and institutional levels. The participants were often stereotyped to be a high-risk group for COVID-19 infection, which contributed to their experience of social segregation, i.e., the separation of groups based on social characteristics. The groups that are being separated would have little or no contact with each other (24).

#### 3.2.1. Community level

##### 3.2.1.1. Being isolated by local Chinese residents

Being secluded was a common experience among the participants during the pandemic because ethnic minorities in Hong Kong were scapegoated for carrying and spreading the virus. Their seclusion experience, i.e., Their experience of being isolated by the mainstream society, was related to the reports of infected cases concerning ethnic minorities, embedded with the longstanding isolation on them before the pandemic:

“An ethnic minority from Dubai got infected in the third wave [of the outbreak], and he spread COVID. Since then, people would stare at me with a very special gaze. People would look at me with an unfriendly gaze when I am on MTR [train]. In the past before COVID, people would avoid having body contact with us; but now their avoidance [has] become more serious, and they would just stand up and walk away from me. They do not want to sit with me…The way they look at us is really very bad, and such a bad gaze is not just from one or two [people]…Whenever I touch anything, they would use alcohol to clean it. I can tell you that these bad experiences are not just my own experiences, but a lot of ethnic minorities here also have similar experiences.” [176, Pakistani, born and educated in Hong Kong]

South Asian minority groups in particular predominantly mentioned their experiences of seclusion during the COVID-19 outbreak:

“There was a time when there was a cluster outbreak [of COVID-19] among the South Asian people. By that time, everyone was scared of us. When we are walking, people would walk 12 feet away. They just keep distance from us like, let’s say 6 feet—no, 12 feet away. They want to keep a lot of distance from us. If we get into the lift, people would not take the same [lift], and they will take another lift instead.” [178, Pakistani, living in Hong Kong for 18 years]

##### 3.2.1.2. Being stereotyped as infectious

The popular experience of seclusion among the participants was closely tied with the public’s stereotypical association between ethnic minorities and COVID-19. As indicated by the participants, ethnic minorities were always perceived as infected by COVID-19 in the eyes of local Chinese residents:

“I can always feel that we are being discriminated [against] by the Chinese. Whenever we go into a lift or anywhere, they would avoid us and turn away their faces. Maybe they are thinking that Indians must have COVID, and they believe that only the Indians would have COVID. Before the COVID [pandemic], such avoidance wasn’t too bad; but now, their avoidance of us has [gotten] much worse. They put the blame on us, and we are always seen as having COVID.” [179, Indian, living in Hong Kong since 2001]

Because of such stereotypes, some participants reported that they were asked to undergo more frequent COVID-19 testing in their workplaces than were their local colleagues:

“All workers who are brown-skin[ned] are instructed to do COVID tests three times a week, but this arrangement is for brown-skin[ned] workers only. The Chinese [workers] do not need to follow this, and they can just get tested in government testing centers once…every 2 weeks.” [186, Pakistani]

To some participants, ethnic minorities are often correlated with negative things in the perceptions of the local Chinese people, and their ethnic minorities are often put in negative expressions. This participant observed that the nicknames of the virus were often attached to ethnic minority groups, making ethnic minorities as if the source of the infection:

“Hong Kong people like using one’s ethnic minority to label something [as] not good. They would label you [as having] the Chinese virus, Japanese virus…or…the Taiwanese virus, Indian virus, Singapore virus, names like these. They put the blame on other ethnic groups… It’s not good, and I strongly feel that it is not appropriate [to] use ethnic minorities to label something bad, and it is not an appropriate word that they use for ethnic minorities.” [194, Indian, living in Hong Kong since 2018]

#### 3.2.2. Institutional level

##### 3.2.2.1. Perceived stigmatization of ethnic minorities by the government

The participants mentioned that they not only experienced seclusion from local Chinese residents but also were stigmatized by the government. For the participants, the government was perceived as having a role in stigmatizing ethnic minority groups because of the constant focus on the ethnic presentation of infected ethnic minorities. Thus, the social situation of ethnic minority groups during the pandemic became more difficult and resulted in them facing greater stigmatization from the local Chinese:

“In the first or second wave [of COVID-19], the infected cases [were] mostly from the Chinese, but the government never said a word about their Chinese race. They would just say the infected person is a male person, a female person. They could have done the same for the third wave; oh, it is a male person, a female person, getting infected; but they didn’t. Why do they have to pick on the race [if] the infected person is an Indian, a Pakistani, or whatever minority? But they would never pick on the Chinese. They [the government officials] don’t have to say those words…They should avoid this kind of picking because in this way, the Chinese [continue to] label us and discriminate [against] us, and this makes our life here more difficult. When a Chinese citizen [is] infected, he’s an infected person. But when a brown person [gets] infected, he must be an Indian or a Pakistani.” [176, Pakistani, born and educated in Hong Kong]

### 3.3. Experiences of the participants before the COVID-19 pandemic

The COVID-19 pandemic was a trigger for the unpleasant experiences of the participants. Their unpleasant experiences were embedded in the existing power differentials between ethnic minority and majority groups before the pandemic, and the pandemic had intensified the further stereotyping and stigmatization of ethnic minority communities. All the participants had experienced segregation and seclusion in different aspects of life before the pandemic.

#### 3.3.1. Community level

##### 3.3.1.1. Segregated social relationships

Being segregated from mainstream society was the most common experience among the participants before the pandemic. Most participants indicated that they had experienced isolation and avoidance from the local Chinese people in different aspects of life before the pandemic, in which the local Chinese people always refused to connect with them:

“It has been many years already and is not a new thing at all. Every time when I traveled on the MTR [train], no one would sit next to me. People would just leave the seat next to me empty, even [when] they [were] standing. If they [could] see [that] I am sitting there, they would move away from me, and the one who [was] sitting close to me would stand up and leave. Even in my neighborhood, neighbors would avoid me too. There are many neighbors, but I can never see them because they would just run back to their flats whenever they realize [that] I [came] out. When I take the lift, they would not go with me but would take another lift.” [189, Indian, living in Hong Kong since 1999]

Such social segregation can pass from generation to generation, which explains the longstanding stigmas toward the participants. This participant shared her experience about how her son was bullied by other local Chinese children before the pandemic, which illustrates the difficulty of ethnic minorities to immerse into the local Chinese community:

“I want my son to make friends, but I don’t know how come the people in Hong Kong and how come their kids can’t be friends with other [skin-] colored people and brown people. Where do these kids get it from? From their parents? But the kids, oh my God, they are such a bully to those with other skin colors. They bully my son a lot just because he is a Pakistani, just because he is brown.” [184, Pakistani]

##### 3.3.1.2. Disadvantaged employment

The participants often experienced difficulties in finding a job before the pandemic. Ethnicity, skin color, and ethnic clothing often caused trouble for them in employment. Although the participants could fulfill the requirement for the job, they were not in the consideration of employment in many cases. In some extreme cases, some ethnic minorities had to give up their traditions and customs for getting an employment:

“They [local employers] do not hire brown-skinned people at all. Every time when I go to a job interview, employers would make use of every excuse to reject you, say [for example] you have to be fluent in Chinese, both speaking and writing. My spoken Chinese is not as fluent as yours, but I can manage daily conversations. [As] for written Chinese, I don’t know why I need to know how to write Chinese for the dishwashing jobs. We do not have much opportunity to learn Chinese, but more than 90% of the jobs would say [that] you have to be fluent in both spoken and written Chinese. Even [if] we have the qualification[s] or we have the efficiency, they would not consider us. For women, it’s more difficult because they are Muslims and have to wear their headscarf. Many Chinese employers do not like people with headscarves. I know some Muslim women give up their headscarf and religion because they want the job.” [177, Pakistani]

Receiving a lower pay was a common experience among the participants before the pandemic, and the situation worsened during the pandemic:

“That [pay discrimination] happened when I applied [as a] part-time waitress. I mentioned [that] my expected salary [was] $60 per hour. The boss didn’t say anything at that time. Then, after I worked for 2 or 3 weeks, my salary was reduced to $55 per hour. I told the boss about my expected salary verbally, and he didn’t refuse, so I assumed [that] it [was] agreed [upon]. In these 2 years of COVID, the unfair treatment toward us regarding salary [became] much worse. My boss always deducts my salary, but other Chinese colleagues can receive the full salary.” [183, Pakistani, born in Hong Kong]

The differential treatment regarding salary also occurred in the government, as mentioned by one participant:

“It was the internal [government] recruitment, and the government needed Urdu, Hindi, or Nepali speakers to help in the compulsory testing centers [for COVID-19]. There was a discrepancy of salary between the local Chinese [workers] and ethnic minorities—and again, we have a lower pay than the Chinese [workers]—though we are all working as interpreters.” [183, Pakistani, born in Hong Kong]

Being among the first ones to be fired was another common experience among the participants before and during the pandemic:

“Such situation[s have] been happening before COVID, but during COVID, the situation has become worse for us. I worked [at] a job for 2 weeks, and then they suddenly said they had enough staff members now, so I was fired immediately. This is not the first time that I [have] experienced this, and it happened already when there was no COVID. I also hear many of these stories because my other South Asian friends were also fired from job[s] because they are brown people. We are always the first ones to get fired.” [183, Pakistani, born in Hong Kong]

##### 3.3.1.3. Racial stereotyping

Many participants believed that whenever a negative event occurred, they, as ethnic minorities, were blamed and scapegoated. The experience of being stereotyped as a criminal before the pandemic was common among the participants:

“I used to do a lot of shopping at a particular shop. After some days, the shop thought that I was stealing, so they asked me to show the things [that I stole]. I showed him the things that I bought and the receipt. I found [this] very insulting. Maybe the shopkeepers reported [me] to the police, and the police came. The police asked me to [step] aside and show them what I [had] stole. They believed that we are stealing. I don’t know why they would [put such a] label on us.” [184, Pakistani]

#### 3.3.2. Institutional level

##### 3.3.2.1. Segregated education

Segregation between children from ethnic minorities and local Chinese children was commonplace according to the participants’ experiences in the educational system of Hong Kong. The participants experienced substantial difficulties in enrolling at Chinese schools. The children of the participants were often required to attend ethnic minority schools even though they fulfilled the academic criteria for admission to local Chinese schools. Presumably this caused remarkable difficulty for the participants to immerse into the local Chinese society and culture since they were young.

“I applied [to] a Band A [the best] secondary school for my daughter, but the EDB [Education Bureau] gave me some ethnic groups’ schools. I asked them why couldn’t my daughter go [to] a Band A Chinese school, as my daughter scored first academically in P.6 [sixth grade at primary school]. However, the EDB staff told me that none of the Chinese schools would take non-Chinese students. They don’t accept non-Chinese students because they worry [that the] reputation of their schools would go down. The more the [number of] non-Chinese [people] going [to] a school, the lower the reputation and the band level would become. I was very upset; it is just racial discrimination… Anyway, my daughter still has to go to an ethnic minority school.” [142, Indian, living in Hong Kong since 1999]

In addition to being segregated from the local schools, some participants mentioned stereotyping in the school environment and stigmatization from their teachers. Their teachers, according to the experiences of some participants in their schools, always stigmatized the ethnic minority students:

“There are some teachers who don’t teach us in EMI [English as the medium of instruction] classes because most ethnic minority students would go to EMI classes, and the teachers [would] kind of judge us as bad, troublesome, and not well-disciplined when compared with the local students in CMI [Chinese as the medium of instruction] classes. Local [Chinese] students of CMI classes are always the focus of care from teachers, and we in the EMI classes are always viewed as bad and naughty students. Many teachers have this impression, oh, this EMI class is going to be rude, [it] is going to be naughty. Not many teachers can accept and understand us. This is rather frustrating because your teachers are looking down on you, so how can you expect [that] others would accept you?” [254, Pakistani, born and receiving secondary school education in Hong Kong]

### 3.4. Effect of seclusion on the participants’ resilience to the pandemic

The prolonged experience of being secluded from the mainstream Hong Kong society among the participants before and during the COVID-19 pandemic remarkably affected their resilience to the pandemic. The participants often relied on fellow ethnic minorities for information and social support, which further segregated the participants from society.

#### 3.4.1. Barriers to obtaining pandemic information

##### 3.4.1.1. Language barrier

The segregation between ethnic minority groups and local Chinese residents caused the participants to experience considerable difficulties in obtaining pandemic-related information. The language barrier was one such difficulty. For those participants who can understand English, the information about COVID-19 was not easily comprehensible due to the gap of English expressions between the institutional channels and their daily usage. For those ethnic minorities who cannot understand English, it is even more difficult for them to obtain COVID-related information, as there was very little pandemic information being disseminated in ethnic languages.

“Although I can understand both English and oral Chinese, still I have to ask my local friends to explain the government information about COVID again. But you cannot always bother your friends, and you can expect [that] they would not be able to tell you 100% of the information. For me, I am educated, and I encounter this problem, so the not-very-well-educated minorities would have an even bigger information gap about COVID. We know much less about COVID than the locals. Also, the English of the government is not easy to understand; it’s not simple, and they are making it so complicated by using those big, big words. I myself [am] educated, but still sometimes I have to read the sentence twice to understand what it’s saying. The words and expression[s] are too formal. Not everyone is educated, so they would not be able to understand those words. This really makes [it] difficult [for us] to understand the government information about COVID. I know many non-Chinese [people] don’t want to read the information because of this.” [176, Pakistani, born and educated in Hong Kong]

As indicated, those participants who had received education in Hong Kong mostly experienced a segregated education experience; either would they have to go to a school designated for ethnic minorities, or would they have to go to a designated class for ethnic minorities. The segregated educational experience of the locally born and educated participants caused them structural difficulties in accessing pandemic-related information, because the syllabus of Chinese language of ethnic minorities schools/classes is different from those in local Chinese schools/classes. The different level of Chinese attainment between ethnic minorities and local Chinese led to disparity in the pandemic information obtained.

“I can speak [in] and read Chinese, but my Chinese is only at an elementary level because I am studying in ethnic [minority] schools. The Chinese that we learnt is much easier, so we cannot read and understand difficult Chinese. I am born and educated in Hong Kong, but my Chinese is not native. Chinese is just like a second language for me, and it is not easy for me to read the COVID information from the government and from the television.” [188, Pakistani, born and educated in Hong Kong]

In many cases, the participants felt that they usually received low-quality information. Besides the segregated education experience that led to their disadvantaged Chinese attainment, there was significant gap in quality and depth between English and Chinese pandemic information as noted by the participants. Pandemic information in Chinese language was often more and deeper, and this was unfavorable for those ethnic minorities who rely on English for pandemic information.

“We cannot get trustable information about COVID because on one hand, it’s [a] language problem because many minorities do not understand English. More detailed and in-depth information is given in Chinese but not in English or in any other ethnic languages. There have been a lot of forums about COVID in Chinese by doctors, which is something much more [in addition] to the government’s information. And a lot more detailed information about COVID is [on] Chinese channels, but not [on] English channels, and there are no ethnic [language] channels. For many of us who don’t know Chinese well, we can only receive much lower [quality] information.” [190, Pakistani, living in Hong Kong since 2011]

##### 3.4.1.2. Lower socioeconomic, education statuses, and social capital as barriers

As suggested by the participants, the relatively low socioeconomic status of most ethnic minorities in Hong Kong has led to them having restricted access to pandemic information. The low education level has also led to low health literacy, which disempowers ethnic minority groups in addressing the pandemic. The social seclusion that the ethnic minorities were experiencing also led to their lack of social capital, which restricted them to obtain resources and information for overcoming the pandemic.

“Most EM [ethnic minorities] don’t know English. My people are mostly the grassroots [very poor people]. They are not educated at all, and they are often in a very, very, very poor situation. They don’t know how to check the COVID information, they can’t read COVID information, and they don’t even know how to prevent themselves from getting infected. They don’t know Chinese, they don’t have many friends who know Chinese, and don’t have many friends who can give them the reliable COVID information.” [240, Pakistani, born in Hong Kong]

#### 3.4.2. Relying on ethnic minorities as social support

Because of the social seclusion of ethnic minorities, the participants were forced to rely on other ethnic minorities for social and material assistance.

“When we encounter anything bad, we would look out for each other, we would tell each other, and we would protect each other because that is the only way for us to deal with it and to survive in this place. Many people do not like us. We are ethnic minorities here, so we even need to be more careful in respecting the rules of [the] people here. When we encounter anything bad, we just avoid it in order not to get into unnecessary fights; and we would stick together and help each other, because no one would help us.” [176, Pakistani, born and educated in Hong Kong]

#### 3.4.3. Sharing incorrect information in social networks

Because of the social seclusion, the participants had to rely on other ethnic minorities to walk through the pandemic. However, other ethnic minorities were also encountering language barrier and limited information access as difficulties. These shared difficulties among ethnic minority groups had made sharing incorrect information about the pandemic with their peers to become a common experience for them.

“I also [have] shared incorrect quarantine information with my family and friends. Last time, [during] the fifth wave [of the COVID-19 outbreak], some people messaged me, ‘people from overseas are allowed to come to Hong Kong.’ I just shared the news through WhatsApp immediately. Then, I found [out] that this information was incorrect. It is difficult for us to get the official pandemic information because most information is in Chinese, and we do not know Chinese. The English version is very rough and not detailed, and many of us do not read English too. Therefore, we mostly rely on [word-of-]mouth information being sent by our ethnic friends. [Although] I know some information may not be true, still sending out [information] first is better than nothing because at least we know something.” [182, Pakistani, living in Hong Kong since 2018]

Because of the barrier to accessing official pandemic information, rumors developed among the ethnic minorities. Some participants indicated that it is not uncommon to see the circulation of inappropriate information about the pandemic within ethnic minority circles:

“My ethnic friends keep telling me that doing the [COVID-19] vaccination is not good. They shared some prank videos saying that the vaccination can make you die. There has been a popular rumor in my community saying that COVID is just a game to fool people. It is about the higher authorities [who] create the virus, create the vaccine, and kill people because they want to reduce the population. Although it sounds pretty nonsensical, many people in my community believe in this rumor, and they refuse to take the vaccine.” [254, Pakistani, born and receiving secondary school education in Hong Kong]

#### 3.4.4. Difficulty conforming to infection control measures

The participants experienced remarkable difficulties in conforming to infection control measures, which was closely linked with their social segregation and low socioeconomic status. In many cases, the participants were unable to purchase infection control materials.

“We couldn’t afford buying the facemasks. I went crazy [about] the high price[s] of masks. Oh my God, the mask prices [are] so crazily high. It is not affordable for us because it is very difficult for us to get a job and not many Chinese [people] are willing to hire us. Even [if] we can be hired, we always have lower salaries than the Chinese [people]. We don’t have money to eat already, so how can we have money to buy masks?” [186, Pakistani, has a history of COVID-19 infection]

#### 3.4.5. Unintentional escape from quarantine

Escaping from quarantine was popular among the participants. However, their escape and failure in conforming to the quarantine order was unintentional in many cases. As mentioned by the participants, the lack of information within ethnic minority circles was a major reason for their escape.

“Many EM [ethnic minorities] ran away from quarantine when they saw the government officials coming to their buildings, because they were scared and did not have any information. Many rumors circulated among [ethnic minority groups], and they believed the government people were coming to catch them. Many EM run away when there is [a] COVID case because they don’t know what would happen to them, and the government appears [to] never share any information with them.” [185, Pakistani, living in Hong Kong since 2011]

In many cases, the lack of support and information that ethnic minorities received because of social seclusion led to them facing difficulties in conforming to the quarantine requirements.

“As an interpreter for fellow ethnic minorities, I always encountered their misunderstandings in [the] quarantine requirement[s] because of [the] language barrier and the lack of social support [that they receive]. Language is a barrier for them to obtain information, leading to their unawareness. They just did not know they had to stay at home during the quarantine, and no one has ever told them. In other scenarios, they had to escape because they were starving, and their children were crying for milk. They did not know who they could turn to for help. They cannot speak Chinese, and many staff in the quarantine hotels cannot understand English. The miscommunication has made so many ethnic minorities [feel] isolated, and it is hard for them to follow the quarantine.” [185, Pakistani, living in Hong Kong since 2011]

#### 3.4.6. Suicidal thoughts

The social seclusion and lack of social support experienced by the participants and their ethnic circles during the COVID-19 pandemic often led to psychological trauma. The mental health of ethnic minorities was often negatively affected and led to some participants experiencing suicidal thoughts. One participant shared how the pandemic led to her and her friends’ depression and suicidal thoughts:

“COVID really makes us crazy. I have some Pakistani friends [who] got infected, and their experiences are all awful. They could not obtain any assistance from the government’s quarantine site. They could not get warm water even though it is a basic human [need]. Because of [the] language [barrier], they could not communicate with the staff at the quarantine site. They felt ignored because they are an ethnic minority. Our experience is really bad here, and we have long been discriminated [against] even before COVID, and a few of my friends developed suicidal thoughts at the quarantine site. My experience is also very bad [during] COVID because my boss fired me. My boss is afraid of me, and no one hires me because I am a Pakistani. I feel really hopeless here.” [187, Pakistani]

## 4. Discussion

Competition theory and conflict theory have been commonly used in understanding the disadvantaged position of ethnic minorities (13–15). As demonstrated by the participants, however, competition theory failed to explain their disadvantaged experiences during the COVID-19 pandemic, as they rarely mention economic scarcity as having a relationship with their disadvantaged encounters. The experiences of our participants align with the conflict theory. Conflict theory explains the social process of how mainstream social groups maintain their advantaged positions by marginalizing minority groups as the dynamics of racial and ethnic relationships in societies (15, 16). By maintaining the negative stereotypes and marginalizing minority groups, mainstream social groups are able to preserve their advantageous position (15). The mainstream social groups are regarded as having higher hierarchy than ethnic minorities, and those who are in the lower hierarchy–ethnic minorities in our case–are often those who experience the negative impacts of this power relationship (23). Those who are in higher position of power are likely to stereotype the powerless, and distribute social resources that favor their own powerful groups (23). The experiences of our participants during the COVID-19 pandemic were predominantly initiated by mainstream stigmatization toward them. They were stereotyped by the mainstream social groups, and were in an unfavorable position for social and health resources during the pandemic. The disadvantaged experiences of our participants in the pandemic should be traced to the embedded social systems, which have put the participants in a segregated position from the mainstream community, making them more vulnerable to the impact of health inequality.

Although our findings echo the conflict theory, our study also adds new perspective of this theory in understanding the disadvantaged experiences of ethnic minorities during the pandemic. The key players as noted in the conflict theory are mainstream social groups in a society, without mentioning about the role of government (15, 16). However, our findings provide a new perspective to this theory that there can be a significant role of government and state policies in marginalizing ethnic minorities, both during and before the pandemic times. As experienced by the participants, the segregated education system for ethnic minorities can reinforce their disadvantaged position in Hong Kong society. This can impose a longstanding impact on the participants in integrating with the mainstream community, and during the pandemic, they were disadvantaged in social support and resources further. Not only did they encounter more difficulties in surviving the pandemic because of their limitation in the local Chinese language and thus their employment and upward social mobility ability, but they also suffered from more severe stigmatization from the mainstream community and from the government. The government and state policies on ethnic minorities, thus, are demonstrated to have a significant role in the experiences of ethnic minorities during the pandemic from our findings.

As experienced by the participants, the education system of Hong Kong has segregated ethnic minorities and mainstream Chinese locals at the institutional level (25). Even for those participants who were born and educated in Hong Kong, they have experienced social segregation since schooling, and they were unable to receive education in mainstream classes and schools in most cases. In worse cases, students of ethnic minorities were stigmatized in schools according to the participants. Their experiences are consistent with the findings of Ku et al. and Loper that have been published almost two decades ago (26, 27). Education in designated schools or classes for ethnic minorities prevented the participants from engaging and interacting with the mainstream locals (26–28), which contributed to structural barriers for them to access local social network. The different education system for ethnic minorities has also led to their language barriers, as their education received in designated schools or classes limited their opportunity to learn Chinese language (26–28), which is one of the official languages in Hong Kong and the major language used in the community. Their lack of opportunity to learn Chinese language in schools limited the participants from interacting with the local mainstream community and from obtaining local information. Coming to the COVID-19 pandemic, such segregation in language and social network as experienced by the participants could then impose negative impact on their resilience in the pandemic. Their lack of opportunity to learn Chinese language in schools also affects other aspects of social determinants, such as having limited their opportunities in further education and employment, which consequently hinders their upward mobility. A vicious cycle of their relatively low socioeconomic status is then resulted, failing them in the access of social and health care resources needed for the pandemic. Such structural disparity and weak social capital for ethnic minorities resulted from social segregation can therefore affect their resilience to a pandemic. As indicated by Parrillo, social segregation implies a lack of exposure and unequal access to resources, such as health, for segregated groups (24), resulting in health inequality. The health inequality as encountered by the participants, thus, is originated from social inequality and wealth inequality (29), which was caused by their marginalization by the mainstream social groups started from education segregation. The experiences of the participants during the COVID-19 pandemic, thus, should be understood as a long social process that was rooted in the power disparity between ethnic minorities and the mainstream social groups at the community and institutional levels.

Because of the segregated education system, the ethnic minorities had limited opportunities to learn Chinese language and to establish social network with the local Chinese community. This can gradually result in social segregation between ethnic minorities and the majority social groups. Social segregation is noted as a form of structural racism that could subsequently affect the health outcomes of ethnic minorities (30). Being noted as a social determinant, racism is noted as closely associated with poorer physical and mental health among ethnic minorities (31, 32), and their poorer mental health is particularly associated with the negative stereotypes attached to their ethnicities (31, 32). In our study, the participants and their ethnic peers also suffered from poor mental health because of the longstanding racial discrimination and stigmatization. Being stereotyped as infectious and subsequently experiencing seclusion in public places were common experiences of the participants during the pandemic. This finding is consistent with those studies that have reported that ethnic minorities are commonly associated with infections and poor mental health because of the intensified racial discrimination from socially mainstream locals during disease outbreaks (33–38). The stereotype of ethnic minorities being infected with and spreading COVID-19 has been constructed by the mainstream locals in Hong Kong at the community and institutional levels during the pandemic. Because the ethnic minority groups in Hong Kong were stereotyped as a high-risk group for infections, the participants experienced various difficulties during the pandemic. In the workplace, they were required to follow more stringent infection control measures than the locals were. Moreover, the government and mass media might have contributed to the stigma toward ethnic minorities during the pandemic because the ethnicity of those infected was often the focal point during reports of outbreaks.

In response to these stigmas at the community and institutional levels, a systematic review shows that ethnic minorities might exhibit distrust toward governments and health care systems during pandemics, which can hinder their accessibility to health care and aggravate the health inequality faced by them (8). In our study, the inaccessibility to proper health care in the pandemic among the participants and their ethnic peers was also profound, as they often exhibited hesitancy and doubt toward the Hong Kong government’s infection control measures, and they commonly relied on their ethnic social networks to overcome the pandemic. This outcome was similar to that of another study in which Asian residents tended to stick with other Asian residents during the COVID-19 pandemic (39). Although ethnic social networks provided important social and material support to the participants, it is important to note that their social networks were unable to offer sufficient pandemic-related information because those who were in their social network were also suffering from the structural disparity and were being secluded from the mainstream society. Consequently, the resilience of ethnic minorities to the pandemic is affected, causing them to be more disadvantaged to adhere to infection control measures or suitably care for their emotional health.

The experiences of the participants during the pandemic were found to be a long social process, as these experiences did not occur suddenly at the pandemic. Rather, their negative experiences were embedded in their experiences before the pandemic. Prior to the pandemic, the participants had been disadvantaged at both community and institutional levels, in aspects such as education, employment, and social network, because of the longstanding stigmatization on them. The pandemic had only made the stigmatization of ethnic minorities to become more intensified and tangible. Because of the preexisting stigmatization and social seclusion of ethnic minorities in Hong Kong, the participants experienced health inequality, which stemmed from social inequality and the power differential between them and the Chinese locals. The participants were disadvantaged in both physical and mental health and they experienced considerable difficulty in receiving accurate pandemic-related information from the mainstream society. To assist the ethnic minorities in overcoming infectious disease outbreaks in future, merely providing short-term material and information assistance to them during an epidemic is barely adequate. Rather, it is vital for policymakers to implement long-term measures that can reduce structural disparity of social determinants in various social systems—such as education, employment, and ethnic minority support—in order to allow a more inclusive social environment for the ethnic minorities. A convergence of health policy together with other social policies is warranted to improve health outcomes for vulnerable social groups (40, 41). Only through strengthening the social capital of the ethnic minorities in various social systems that can empower them to have a higher resilience in future infectious disease outbreaks.

### 4.1. Limitations

The participants were sampled from a university, a non-governmental organization, and a district councilor’s office. Those ethnic minorities who were not in touch with these three sampling sites were not in the sampling pool. Future studies with more sampling sites may add confidence in the findings.

## 5. Conclusion

Although studies have found that ethnic minorities are often disadvantaged during disease outbreaks, the reasons for their experiences are not well documented. Moreover, how their disadvantaged experiences during disease outbreaks are related to the embedded and longstanding stigma that they receive is poorly understood. The present study therefore investigated the experiences of the individuals of South Asian and African origin living in Hong Kong during the COVID-19 pandemic, and how their unpleasant experiences were embedded in the longstanding stigma of ethnic minorities in Hong Kong. As experienced by the participants, being isolated and being stereotyped as infectious were popular encounters during the pandemic. These encounters, though, did not happen suddenly during the pandemic, but they were embedded in the longstanding stigmatization against ethnic minorities in pre-pandemic times. Furthermore, the segregated education system of ethnic minorities has created barriers for them to integrate with the mainstream local Chinese community, making them difficult to access to local Chinese language and local Chinese social network. These barriers, as a result, negatively affected their resilience when living and coping with the pandemic.

## Data availability statement

The raw data supporting the conclusions of this article will be made available by the authors, without undue reservation.

## Ethics statement

The studies involving human participants were reviewed and approved by Human Subjects Ethics Subcommittee of the Hong Kong Polytechnic University before the study began (ID: HSEARS20200827001-04). The patients/participants provided their written informed consent to participate in this study.

## Author contributions

DS, YC, and JS contributed to the study design. DS and YC contributed to the data collection. JS contributed to the data collection training, data analysis, interpretation, and writing. DS and YC provided critical suggestions in the data analysis and interpretation, and engaged in the critical revision of the manuscript. All authors have read and approved the final manuscript.

## Funding

The authors disclosed receipt of the following financial support for the research, authorship, and/or publication of this article: This work was undertaken with support from the Health and Medical Research Fund [Commissioned Research on the Novel Coronavirus Disease (COVID-19)], Food and Health Bureau, The Government of the Hong Kong Special Administrative Region [grant number: COVID190217].

## Conflict of interest

The authors declare that the research was conducted in the absence of any commercial or financial relationships that could be construed as a potential conflict of interest.

## Publisher’s note

All claims expressed in this article are solely those of the authors and do not necessarily represent those of their affiliated organizations, or those of the publisher, the editors and the reviewers. Any product that may be evaluated in this article, or claim that may be made by its manufacturer, is not guaranteed or endorsed by the publisher.
